# Enhancement of dissolution and oral bioavailability of lacidipine via pluronic P123/F127 mixed polymeric micelles: formulation, optimization using central composite design and *in vivo* bioavailability study

**DOI:** 10.1080/10717544.2017.1419512

**Published:** 2017-12-23

**Authors:** Ahmed R. Fares, Aliaa N. ElMeshad, Mohamed A. A. Kassem

**Affiliations:** ^a^ Department of Pharmaceutics and Industrial Pharmacy, Faculty of Pharmacy, Cairo University Cairo Egypt

**Keywords:** Lacidipine, central composite design, pluronics, polymeric micelles, dissolution rate, bioavailability study

## Abstract

This study aims at preparing and optimizing lacidipine (LCDP) polymeric micelles using thin film hydration technique in order to overcome LCDP solubility-limited oral bioavailability. A two-factor three-level central composite face-centered design (CCFD) was employed to optimize the formulation variables to obtain LCDP polymeric micelles of high entrapment efficiency and small and uniform particle size (PS). Formulation variables were: Pluronic to drug ratio (A) and Pluronic P123 percentage (B). LCDP polymeric micelles were assessed for entrapment efficiency (EE%), PS and polydispersity index (PDI). The formula with the highest desirability (0.959) was chosen as the optimized formula. The values of the formulation variables (A and B) in the optimized polymeric micelles formula were 45% and 80%, respectively. Optimum LCDP polymeric micelles had entrapment efficiency of 99.23%, PS of 21.08 nm and PDI of 0.11. Optimum LCDP polymeric micelles formula was physically characterized using transmission electron microscopy. LCDP polymeric micelles showed saturation solubility approximately 450 times that of raw LCDP in addition to significantly enhanced dissolution rate. Bioavailability study of optimum LCDP polymeric micelles formula in rabbits revealed a 6.85-fold increase in LCDP bioavailability compared to LCDP oral suspension.

## Introduction

Lacidipine (LCDP) is a potent 1,4-dihydropyridine calcium channel blocker. LCDP lowers the blood pressure by dilating the peripheral arterioles and reducing peripheral vascular resistance. LCDP is used in the treatment of hypertension and atherosclerosis in addition to its antioxidant activity (Hanes & Weir, 2005; ElKasabgy et al., 2014). LCDP suffers from low oral bioavailability about 10% (range 3–59%) due to extensive hepatic first-pass metabolism by cytochrome P450 3A4 (CYP3A4). In addition, LCDP is a highly lipophilic BCS class II drug with poor aqueous solubility which contributes to its limited bioavailability (Basalious et al., 2010; Gannu et al., 2010; Wu et al., 2015; Zhao et al., 2015; Darekar et al., 2016).

Different approaches have been used to improve LCDP solubility and dissolution rate limited absorption and in turn its oral bioavailability including solid dispersions (Mukharya et al., 2012; Sun et al., 2016), lipotomes (ElKasabgy et al., 2014), microemulsion based gels (Gannu et al., 2010), nanosuspensions (Dinda & Pand, 2014; Zhao et al., 2015; Kassem et al., 2016) and self-nanoemulsifying drug delivery systems (Basalious et al., 2010).

Polymeric micelles could be another good strategy to improve LCDP solubility and oral bioavailability. Polymeric micelles are nanoscopic core/shell structures formed by self-assembly of amphiphilic block copolymers consisting of both hydrophilic and hydrophobic monomer units. The self-assembly of polymeric micelles starts when the concentration of the polymers exceeds the critical micelle concentration (CMC). The hydrophobic part of the block copolymer forms the inner core which encapsulates the poorly water-soluble drug, whereas the outer shell of the hydrophilic block of the copolymer protects the drug from the aqueous environment (Rangel-Yagui et al., 2005; Aliabadi et al., 2008; Kedar et al., 2010).

Polymeric micelles formation can be an effective alternative to increase the solubility and bioavailability of hydrophobic drugs as: (a) they are more stable than the surfactant counterparts, with lower CMC and slower rate of dissociation, (b) their size is normally around 5–100 nm which help to evade scavenging by the mononuclear phagocytic system in the liver leading to longer blood circulation time, and (c) they prevent the precipitation of poorly water soluble drugs in the fluids of the GI tract (Kataoka et al., 2001; Gaucher et al., 2005; Rangel-Yagui et al., 2005; Kedar et al., 2010; Lu and Park, 2013).

Lacidipine polymeric micelles help to overcome the disadvantages of other approaches used to improve LCDP solubility. Nanosuspensions approach in many instances needs a high energy input which increases the production cost. Moreover, extra steps are required to remove any residual organic solvent above maximum acceptable levels. Inability to control the droplet size is a major problem in nanoemulsions approach. In addition, nanoemulsions show reduced stability where flocculation and coalescence often occurs upon storage (Lu and Park, 2013).

Pluronics are amphiphilic triblock copolymers made of hydrophilic poly ethylene oxide (PEO) and hydrophobic poly propylene oxide (PPO) blocks (PEO–PPO–PEO). A wide array of Pluronics is available depending on their molecular characteristics obtained by varying the PPO/PEO ratio and/or the molecular weights such as F127 (PEO100–PPO69–PEO100) and P123 (PEO20–PPO65–PEO20) (Kedar et al., 2010; Kulthe et al., 2011). Due to their solubilization effect and inhibition of P-glycoprotein (P-gp) efflux, Pluronic polymeric micelles increase the bioavailability and circulation time of poorly water soluble drugs (Abdelbary and Makhlouf, 2014). Several studies have been conducted on the solubilization and delivery of hydrophobic drugs using Pluronic polymeric micelles (Alakhov et al., 2004; Yong et al., 2005; Zhang et al., 2011b; Zhao et al., 2012; Chen et al., 2013; Abdelbary & Makhlouf, 2014).

Central composite face-centered design (CCFD) is a second order design which requires only three levels of each factor. CCFD enables the study of the effects of each factor and their interactions over the responses using fewer experimental runs compared to a full-factorial design. In addition CCFD can be used for prediction and optimization of the responses. A two-factor three-level CCFD consists of four factorial points, four axial points and replicated center points. Factorial points participate in estimating the linear terms and two factor interactions, axial points participate in estimating the quadratic terms and the center points are repeated to estimate the pure experimental uncertainty at the factor levels (Montgomery, 2001; El-Mahrouk et al., 2014; El Mahrouk et al., 2014).

In the present study, LCDP loaded polymeric micelles composed of Pluronic F127 (F127) and Pluronic P123 (P123) were prepared by the thin film hydration technique in order to overcome LCDP solubility limited oral bioavailability. A two-factor three-level CCFD was used to study the influence of different variables on each studied response and to find an optimized formula. The independent variables selected were: Pluronic to drug ratio (A) and P123 percentage (B). Entrapment efficiency (EE%) (*Y*_1_), particle size (PS) (*Y*_2_), and polydispersity index (PDI) (*Y*_3_) were chosen as dependent variables. The optimum LCDP polymeric micelle formula was physically characterized using transmission electron microscopy (TEM). The saturation solubility and dissolution rate of the optimum LCDP polymeric micelle formula were also determined and compared to raw LCDP. The optimum formula was further evaluated for its *in vivo* performance in rabbits compared to LCDP oral suspension.

## Materials and methods

### Materials

Lacidipine was kindly supplied by Egyphar Co. (Cairo, Egypt). Pluronic F127 (F127), Pluronic P123 (P123), tertiary butyl methyl ether, acetonitrile (HPLC grade) and formic acid were purchased from Sigma-Aldrich Chemical Co. (St. Louis, IL, USA). Hydrochlorothiazide (internal standard), was kindly supplied by Genuine Research Center (Cairo, Egypt). All other chemicals were of analytical grade and used as received.

### Experimental design

A two-factor, three-level (3^2^) CCFD was employed to statistically optimize the formulation variables of LCDP polymeric micelles preparation. Generation and evaluation of the experimental design was carried out using the Design Expert^®^ software (Version 7; Stat-Ease Inc., Minneapolis, MN, USA). According to the followed CCFD, 11 formulae were prepared. Each formula was performed twice in two separate replicates giving a total of 22 runs. The independent variables were: Pluronic to drug ratio (A) and P123 percentage in the total Pluronic mixture (B). The levels of each factor were designated as (−1, 0, +1) and their corresponding actual values are shown in Table 1. The composition of the 11 formulae of the 3^2^ CCFD listed in standard order are shown in Table 2. Analysis of variance (ANOVA) was carried out to estimate the significance of model and term. Probability *p*-values (*p* < .05) denoted significance. EE% (*Y*_1_), PS (*Y*_2_), and PDI (*Y*_3_) were chosen as dependent variables.

**Table 1.** Independent variables and respective levels in the 3^2^ CCFD for LCDP polymeric micelles preparation, model summary statistics of quadratic model, constrains for optimization and factors levels for optimized LCDP polymeric micelles formula and their predicted and observed values.

		Levels of variables					
Factors (independent variables)		Low (−1)	Medium (0)	High (+1)		Optimized level	
A: Pluronic to drug ratio^a^		10	30	50		45	
B: P123 percentage (% w/w)		10	50	90		80	
Responses	*r*^2^	Adjusted *r*^2^	Prediction *r*^2^	Constrains	Predicted	Observed	95% prediction interval
*Y*_1_: Entrapment efficiency (%)	0.9162	0.8900	0.8247 (0.8311)^b^	Maximize	100	99.23	81.06–123.26
*Y*_2_: Particle size (nm)	0.8650	0.8229	0.7200	Minimize	19.88	21.08	9.74–30.02
*Y*_3_: PDI	0.9053	0.8757	0.8257 (0.8366)^b^	Minimize	0.097	0.11	0.039–0.160

^a^ The levels of variable (A) are 10:1, 30:1 and 50:1. Since the drug is always designated in the ratio as “1”, so the levels of variable (A) were written as 10, 30 and 50.

^b^ Reduced model prediction *r* ^2^.

**Table 2.** Composition of the 3^2^ CCFD and the average EE%, PS and PDI for the prepared LCDP polymeric micelles.

	Factors levels in actual values				
Formula	Pluronic to drug ratio	P123 percentage (%)	EE% + SD (%)^a^	PS + SD (nm)^a^	PDI + SD^a^
M1	10	10	7.27 ± 0.47	58.73 ± 1.56	0.23 ± 0.01
M2	50	10	98.72 ± 0.72	26.34 ± 0.09	0.14 ± 0.01
M3	10	90	79.22 ± 1.00	26.83 ± 0.96	0.29 ± 0.03
M4	50	90	99.55 ± 0.18	20.03 ± 0.21	0.08 ± 0.02
M5	10	50	64.28 ± 0.98	26.07 ± 1.07	0.32 ± 0.04
M6	50	50	96.69 ± 0.77	23.46 ± 0.29	0.14 ± 0.02
M7	30	10	97.35 ± 0.25	29.93 ± 0.76	0.18 ± 0.01
M8	30	90	98.15 ± 0.89	20.65 ± 0.20	0.15 ± 0.01
M9	30	50	97.75 ± 0.16	23.13 ± 0.16	0.15 ± 0.03
M10	30	50	97.23 ± 0.41	23.12 ± 0.53	0.14 ± 0.01
M11	30	50	99.43 ± 0.73	23.74 ± 0.95	0.13 ± 0.03

^a^ All measurements are done in triplicates.

### Preparation of LCDP polymeric micelles by thin film hydration technique

A mixture of LCDP (20 mg) and different weights of F127 and P123 according to the abovementioned design was dissolved in acetone (10 mL) in a round bottom flask (500 mL). The solvent was subsequently evaporated at 60 °C under reduced pressure (1 mmHg) using a rotary vacuum evaporator (Stuart RE300; Bibby Scientific LTD, Staffordshire, UK) at 120 rpm to obtain a thin dry film of LCDP/F127/P123 on the flask wall. The resulted film was then hydrated using 10 mL distilled water under normal pressure for 1 h. Unincorporated drug aggregates were removed by filtration through 0.45 μm membrane filter and LCDP polymeric micelles dispersion was obtained (Abdelbary & Tadros, 2013; Chen et al., 2013; Abdelbary & Makhlouf, 2014).

### Determination of EE%

Entrapment efficiency of LCDP in polymeric micelles was determined from the micellar dispersion obtained after separation of the unincorporated drug by filtration through 0.45 μm membrane filter. EE% of LCDP was analyzed spectrophotometrically at *λ*_max_ 286 nm (Shimadzu UV-1601 PC, Kyoto, Japan) after appropriate dilution with ethanol. LCDP concentration was determined by comparing the absorbance of the filtered solution to a preconstructed drug calibration curve in 1:1 mixture distilled water/ethanol (*R* ^2^ = 0.9994, *n* = 3). Each experiment was carried out in triplicate and the mean value was deduced. The EE% was calculated according to equation (1) (Zhao et al., 2012; Abdelbary & Tadros, 2013):

(1) $$\text{EE}\%=\frac{\text{Weight}\;\text{of}\;\text{drug}\;\text{in}\;\text{micelles}}{\text{Weight}\;\text{of}\;\text{drug}\;\text{used}\;\text{in}\;\text{micelles}\;\text{preparation}}\times 100$$

### PS analysis of LCDP polymeric micelles

The PS and PDI were determined after appropriate dilution with deionized water by dynamic light scattering (DLS) at 25 °C using Zetasizer Nano ZS (Malvern Instrument Ltd., Worcestershire, UK). DLS analyzes the fluctuations in light scattering due to the Brownian motion of particles. All measurements were performed in triplicate (Zhang et al., 2011a; Abdelbary & Makhlouf, 2014).

### Formulation optimization

The optimized LCDP polymeric micelle formula was obtained using the Design Expert^®^ software by applying constraints on the EE%, PS and PDI, as shown in Table 1. The suggested optimized LCDP polymeric micelle formula was then prepared and evaluated in triplicate to check the validity of the calculated optimal formulation factors and predicted responses given by the software. The observed responses are considered acceptable if they lie within the 95% prediction interval represented in Table 1. For further characterization, the freshly prepared optimal LCDP polymeric micelles formula was lyophilized for 24 h with a condenser temperature of −45 °C and under vacuum of 7 × 10^−2^ mbar (Novalyphe-NL 500, Halprook, NY, USA).

### Characterization of the optimal LCDP polymeric micelles

#### Determination of EE% and PS analysis

Entrapment efficiency, particle size and polydispersity index of optimal LCDP polymeric micelles before and after lyophilization was determined as previously described.

### Determination of CMC

The CMC of optimum P123/F127 mixed micelles formula [composed of 80% P123 & 20% F127] in addition to the CMC of pure F127 and P123 micelles in distilled water were determined using the iodine (I_2_) UV spectroscopy method (Wei et al., 2009). Amounts of 0.5 g I_2_ and 1 g potassium iodide (KI) were dissolved in 50 ml distilled water to prepare the KI/I_2_ standard solution. Thirty samples of P123/F127, F127 and P123 solutions with concentrations ranging from 0.00001% to 0.1% were prepared. An aliquot of 100 μl of KI/I_2_ standard was added to each Pluronic solution. Before measurement, the mixtures were incubated for 12 h in a dark place at room temperature. The UV absorbance of different P123/F127, F127 and P123 concentrations at 366 nm was measured (Shimadzu UV-1601 PC, Kyoto, Japan). Experiments were done in triplicate. For CMC determination, the absorbance was plotted against the logarithm of Pluronic concentrations. The Pluronic concentration, at which sharp increase in absorbance is observed, corresponds to the CMC.

#### Transmission electron microscopy

The morphology of optimum LCDP polymeric micelles formula was examined by TEM. One drop of LCDP micellar dispersion was placed onto a carbon-coated copper grid, leaving a thin liquid film which was negatively stained by 2% phosphotungstic acid. The sample was air dried slowly. The film was then examined under Joel TEM (Jeol; JEM-1230, Tokyo, Japan) and photographed (Abdelbary & Tadros, 2013; Abdelbary & Makhlouf, 2014).

#### Scanning electron microscopy

The surface morphology of the optimum lyophilized polymeric micelles was examined by scanning electron microscopy (SEM). Lyophilized sample was fixed with double-sided adhesive tape on the SEM sample holder and coated under an argon atmosphere with gold using a sputter coater (Edwards S-105 A, England, UK) to achieve a film of 150 A^o^ thickness. The samples were then examined using SEM (Jeol; JEM-2100, Tokyo, Japan).

#### Saturation solubility in distilled water and 0.1 M HCl(pH = 1.2)

The saturation solubility of raw LCDP, lyophilized LCDP suspension [composed of 20 mg LCDP, 10%w/v acacia, 20%v/v glycerin in 10 mL water] and lyophilized LCDP polymeric micelles in distilled water and 0.1 M HCl (pH = 1.2) was determined according to Tenjarla et al. method. An excess quantity of either raw LCDP, lyophilized LCDP polymeric micelles or lyophilized LCDP suspension was added to either 3 mL distilled water or 0.1 M HCl in screw capped glass vials. The vials were placed in a shaking water bath (Model 1083; GLF Corp., Burgwedel, Germany) maintained at 50 rpm at 37 °C for 24 h. Then, the solutions were filtered through a 0.45 µm Millipore filter. The amount of the drug dissolved was analyzed spectrophotometrically at *λ*_max_ 286 nm (Shimadzu UV-1601 PC, Kyoto, Japan). Each experiment was carried out in triplicate and the mean value was deduced.

#### *In vitro* dissolution rate study

The dissolution rate of LCDP suspension as well as the lyophilized LCDP polymeric micelles was performed using a USP standard paddle apparatus (USP Dissolution Tester, Varian, model VK7000, USA). The vessel was filled with 100 mL 0.1 M HCl (pH 1.2) at 37 ± 1 °C and stirred at 100 rpm. Accurately weighed samples of lyophilized LCDP polymeric micelles or 2 mL of LCDP suspension containing the equivalent of 4 mg LCDP were dispersed in the dissolution medium. Aliquots each of 2 mL were withdrawn from the dissolution medium through 0.22 μm Millipore membrane filter at 5, 10, 15, 20 and 30 min time intervals and replaced by equal volume of fresh 0.1 M HCl kept at the same temperature. The concentration of the dissolved drug was measured spectrophotometrically at *λ*_max_ 286. The experiments were done in triplicate and the average ± SD was calculated. Non-sink conditions were utilized to distinguish the improvement achieved in the dissolution rate (Basalious et al., 2010; Xia et al., 2010; ElKasabgy et al., 2014).

### *In vivo* bioavailability study in rabbits

#### Study design

This study was carried to compare the bioavailability of the optimum LCDP polymeric micelles formula to the oral LCDP suspension previously prepared following administration of single dose of 4 mg each, using a non-blind, two-treatment, two-period, randomized, crossover design. The study was approved by the Cairo university research ethics committee (serial number of the protocol: PI(E) 1003).

Six male healthy albino rabbits (each weighing between 2 and 2.5 kg) were randomly divided into two groups (three rabbits per each group). A simple cross over design was applied on two phases where the rabbits in each group received a single 2 mL oral dose (4 mg LCDP) of one of the tested formulae in each phase through a feeding tube. In phase I, rabbits of group one received the test (treatment A) and those of group two received the LCDP oral suspension (treatment B) which was considered as a standard. A washout period of two weeks separated the two phases. On the second phase, the reverse of randomization took place.

#### Sample collection

Blood samples were withdrawn from marginal ear vein of each rabbit into heparinized tubes at the following time points: 0 (pre-dose), 0.5, 1, 2, 4, 6, 8, 12, 24, 48 and 72 h after administration of each treatment. Plasma was immediately separated by centrifugation at 3500 rpm for 10 min. The plasma was then transferred directly into 5 mL plastic tubes and stored frozen at −20 °C pending drug analysis.

#### Sample preparation

All frozen plasma samples were thawed at ambient temperature. A liquid–liquid extraction procedure was used. Plasma samples (0.5 mL) were placed in 10 mL glass tubes, and 50 μL of hydrochlorothiazide solution (100 ng/mL) as internal standard (IS) was added to each and vortexed for 30 s. About 4 mL of tertiary butyl methyl ether was then added, and samples were then vortexed for 3 min. The tubes were then centrifuged for 10 min at 4000 rpm. The upper organic phases were then transferred to clean glass tubes, and evaporated to dryness using centrifugal vacuum concentrator (Eppendorf 5301, Germany) at 40°C. Dry residues were then reconstituted with 200 μL of mobile phase and vortex mixed for 1 min, and 20 μL was injected using the autosampler.

#### LC/MS/MS assay of LCDP

A sensitive, selective and accurate LC/MS/MS method was adopted for the analysis of LCDP plasma concentrations. The LC/MS/MS method was previously developed and validated by ElKasabgy et al. (2014).

#### Pharmacokinetic and statistical analysis

Plasma concentration–time data of LCDP was analyzed for each rabbit by non-compartmental pharmacokinetic models using KineticaTM2000 softare (ver3, InnaPhase Corporation, USA). The maximum plasma concentrations (*C*_max_, ng/mL) and the time to reach them (*t*_max_, h) were obtained from the individual plasma concentration–time curves. The area under the curve from zero to 72 h (AUC_(0–72)_, ng h/mL) and to infinity (AUC_(0–∞)_, ng h/mL), were calculated using the linear trapezoidal rule. In addition, the terminal elimination rate constant (*λ*_z_, h^−1^) was calculated using linear regression to the terminal portion of the ln concentration–time curve and the elimination half-life (*t*_1/2_) was also calculated. Results were expressed as mean values of six rabbits ± SD. To investigate the statistical significance among groups, two way analysis of variance test (ANOVA) was used to compare the *C*_max_, AUC_(0–72)_, AUC_(0−∞)_, *λ_z_* and *t*_1/2_ of treatments A and B via SPSS 17.0 software (SPSS Inc., Chicago, IL, USA). Nonparametric Signed Rank Test (Mann–Whitney’s test) was used to compare the medians of *t*_max_ for treatments A and B using the same software. A *p* < .05 was considered statistically significant.

## Results and discussion

### Statistical analysis of the 3^2^ CCFD

The responses, *Y*_1_: EE%, *Y*_2_: PS and *Y*_3_: PDI were analyzed individually and fitted to linear, two factor interaction (2FI) and quadratic models using linear regression. The model with the highest adjusted *r* ^2^ and prediction *r* ^2^ was the one of choice. Model reduction by removing any non-significant model terms that were not needed to support hierarchy was adopted to improve the chosen model (Gonnissen et al., 2008).

#### EE% model analysis

Entrapment efficiency of LCDP was measured preliminarily in micelles prepared from only F127 or P123 using the highest Pluronic to drug ratio in the design (50:1). Pure F127 or P123 micelles, even by this high Pluronic to drug ratio, was unable to successfully entrap LCDP and achieve a good EE%. F127 micelles showed an EE% of only 27.36% while P123 micelles had an EE% of 35.28%.

Entrapment efficiency of LCDP polymeric micelles ranged from 7.27 ± 0.47 to 99.55 ± 0.18% as shown in Table 2. Quadratic model was chosen for EE% since it has the highest adjusted and prediction *r* ^2^ according to the EE% model summary statistics represented in Table 1. Significant terms of the quadratic model on EE% was identified using ANOVA test, where only model terms with *p* < .05 were considered statistically significant. Higher prediction *r* ^2^ was achieved through model reduction by removing the non-significant model terms as shown in Table 1. The final equation relating different factors and interactions for EE% in terms of coded variables is as follows:

(2) $$\mathrm{EE\%=97}\mathrm{.98+24}\mathrm{.03}A\mathrm{+ 12}\mathrm{.26}B-\mathrm{17}\mathrm{.78}AB\mathrm{- 23}\mathrm{.69}A^{2}$$

ANOVA analysis indicated that Pluronic to drug ratio and P123 percentage had a significant quadratic effect on EE%. Increasing the Pluronic to drug ratio was associated with a significant increase in the EE% (*p* < .0001). It was observed that at low Pluronic to drug ratio, obvious drug precipitation and lower EE% was achieved. Drug precipitation could be due to the saturation of the inner core of the micelles by the drug at low Pluronic to drug ratios when the amount of the drug was higher relative to the Pluronic amount in the formula. So it could be assumed that mixed micelles may enhance the solubility and EE% of poorly soluble drugs to a certain limit, after which increasing the drug amount and decreasing the Pluronic to drug ratio leads to drug precipitation (Mu et al., 2010).

In addition, increasing the P123 percentage was associated with a significant increase in the EE% (*p* < .0001). P123 is a relatively hydrophobic Pluronic with long PPO and short PEO chains, while F127 is a hydrophilic one with a high ratio of PEO/PPO. Both P123 and F127 have nearly the same length of the hydrophobic moiety which is (PPO: 65) and (PPO: 69) respectively. So, the proper ratio between the two Pluronics is critical for the formation of drug loaded mixed micelles. So, increasing the P123 percentage is expected to increase the EE% due to the better solubilization capacity of the hydrophobic P123 compared to F127 (Wei et al., 2009).

**Figure 1.**
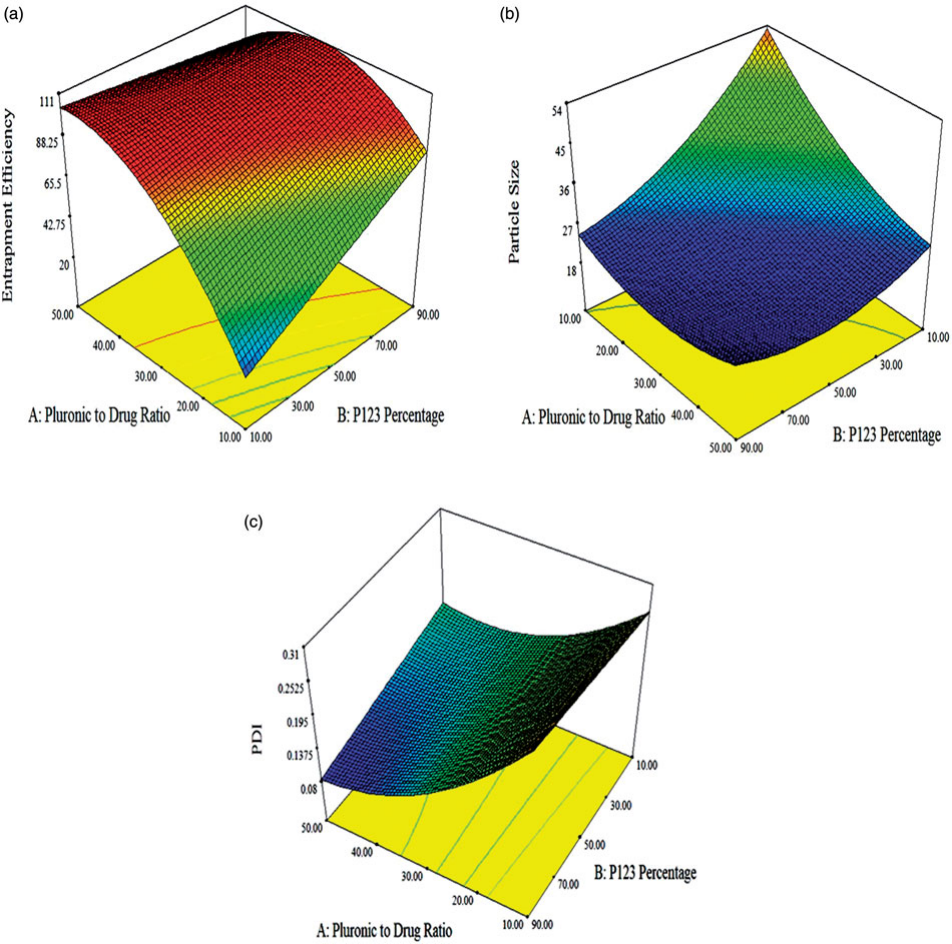
Response surface plot for the effect of Pluronic to drug ratio and P123 percentage on: (a) EE%, (b) PS and (c) PDI.

Also according to Allen et al. (1999), the compatibility between the loaded drug and the core forming block affects the EE%. The poorly water soluble LCDP is expected to have higher affinity to the more hydrophobic P123 than the hydrophilic F127. This means that the compatibility between LCDP and the core of the mixed polymeric micelles is better in the presence of high P123 percentage. So, increasing the LCDP EE% is highly dependent on increasing the P123 percentage. Figure 1(a) illustrates the response surface plot for the effect of Pluronic to drug ratio and P123 percentage on EE%.

#### PS model analysis

Various studies showed that polymeric micelles can be absorbed intact to the systemic circulation after oral administration by pinocytosis as a major route. The PS of polymeric micelles affects the rate and extent of this absorption (Gaucher et al., 2010). In addition, to achieve longer systemic circulation, the prepared mixed micelles must be small enough to evade the recognition by the RES. Normally, particles smaller than 10 nm are filtered through the kidney and particles larger than 100 nm are captured by the liver. So, the ideal size is between 10 and 100 nm (Yu et al., 2010). As shown in Table 2, PS of LCDP polymeric micelles ranged from 20.03 ± 0.21 to 58.73 ± 1.56 nm, suggesting they could be good drug carriers able to escape the RES and extending the drug effect in the body. The PS of LCDP polymeric micelles was small compared to PS of other different reported LCDP nanosystems such as, LCDP nanocrystals in our previous study (273.21 nm) (Kassem et al., 2016), LCDP nanocrystals prepared by Zhao et al. (2015) (714 nm) and LCDP lipotomes prepared by Elkasabgy et al. (2014) (305.52 nm).

Particle size model summary statistics represented in Table 1 showed that the quadratic model has the highest adjusted and prediction *r* ^2^, accordingly, it was the model of choice for PS. The final equation relating different factors and interactions for PS in terms of coded variables is as follows:

(3) $$\mathrm{PS =22}\mathrm{.01-6}\mathrm{.97}A\mathrm{- 7}\mathrm{.91}B\mathrm{+ 6}\mathrm{.4}AB\mathrm{+ 4}\mathrm{.73}A^{2}\mathrm{+ 5}\mathrm{.25}B^{2}$$

ANOVA analysis showed that Pluronic to drug ratio and P123 percentage had a significant effect on PS. Increasing both factors was associated with a significant decrease in the PS (*p* < .0001). Response surface plot for the effect of Pluronic to drug ratio and P123 percentage on PS is illustrated in Figure 1(b).

Decreasing the Pluronic to drug ratio and increasing the drug amount relative to the Pluronics may increase the PS due to the enlargement of the polymeric micelles hydrophobic core region by the entrapment of higher drug amount in less number of polymeric micelles (Kulthe et al., 2011). Also, increasing the drug amount may lead to the adsorption of drug molecules on the outer shell of the micelles leading to PS enlargement (Rupp et al., 2010).

In a previous study, Abdelbary and Tadros (2013) stated that increasing the P123 concentration significantly reduced the PS of olanzapine loaded micelles from 36.01 to 18.97 nm. This may be attributed to the conversion of the micelles to a smaller mixed micelles structure. In addition, the larger molecular weight F127 accounts for colloidal steric stabilization by its large hydrophilic heads (Wei et al., 2009), so decreasing its amount may lead to PS reduction.

#### PDI model analysis

Polydispersity index was studied to optimize a formula with the least PS variability. A PDI value less than 0.1 represents a highly homogenous PS distribution, whereas, high PDI values suggest a broad PS distribution (Gaumet et al., 2008). The PDI of prepared LCDP polymeric micelles ranged from 0.08 ± 0.02 to 0.32 ± 0.04 as shown in Table 2 indicating an acceptable homogenous PS distribution. Quadratic model had the highest adjusted and prediction *r* ^2^ as represented by the PDI model summary statistics in Table 1. Model reduction led to higher prediction *r* ^2^. The final equation relating different factors and interactions for PDI in terms of coded variables is as follows:

(4) $$\mathrm{PDI =0}\mathrm{.15-0}\mathrm{.083}A\mathrm{-0}\mathrm{.0075}B\mathrm{-0}\mathrm{.026}AB\mathrm{+0}\mathrm{.05}A^{2}$$

ANOVA analysis indicated that increasing Pluronic to drug ratio was associated with a significant decrease in the PDI (*p* < .0001). On the other hand, P123 percentage had a non-significant effect on PDI. Figure 1(c) illustrates the response surface plot for the effect of Pluronic to drug ratio and P123 percentage on PDI. Increasing the Pluronic to drug ratio decreased the PS and favored homogeneous PS distribution. This narrow size distribution implied the co-micellization of the two copolymers (Mu et al., 2010).

### Formulation optimization

After applying constrains on EE%, PS and PDI, the formula with an overall desirability of 0.959 was chosen by the Design Expert^®^ software as the optimized formula. Factors levels for the optimized formula are shown in Table 1.

### Characterization of the optimal LCDP polymeric micelles

#### EE% and PS analysis

The suggested LCDP polymeric micelles formula was prepared and evaluated. The observed EE%, PS and PDI lie within the 95% prediction interval calculated demonstrating the validity of the optimization and prediction processes, as shown in Table 1.

Entrapment efficiency, particle size and polydispersity index of optimal LCDP polymeric micelles after lyophilization were determined and compared to those observed before lyophilization. This was done to ensure that the prepared LCDP polymeric micelles had the same properties after redispersion. After redispersion, the EE% was 98.31 ± 0.84%, the PS was 22.28 ± 0.31 nm and the PDI was 0.192 ± 0.02 which was statistically non-significant from the observed values listed in Table 1.

### CMC

Critical micelle concentration is a major parameter in determining the *in vitro* and *in vivo* stability of polymeric mixed micelles. Low CMC values of P123/F127 mixed micelles demonstrate the high stability of P123/F127 micelles upon dilution. I_2_ was used as a hydrophobic probe, where the solubilized I_2_ prefers to reside in the hydrophobic microenvironment of P123/F127 copolymers. To keep the I_2_ saturated concentration in water, I_3_^-^ is converted to I_2_ from the excess KI in the solution. By plotting the I_2_ absorbance as a function of the logarithm of P123/F127 concentrations as shown in Figure 3(a), the CMC of optimum P123/F127 mixed micelles formula was determined to be 0.0056%. This relatively low CMC value of P123/F127 mixture indicates the high stability of their mixed micelles and their ability to maintain integrity even after extreme dilution by the body fluids. In addition, the CMC values of pure F127 and P123 were measured. The CMC of F127 and P123 were in agreement with the previously determined CMC values in literature where the CMC of F127 and P123 were 0.0035% and 0.0071%, respectively (Kabanov et al., 2003; Wei et al., 2009). The CMC of P123/F127 mixture had an intermediate value between pure F127 and P123. This may be attributed to the hydrophobic interactions between the two Pluronics PPO chains (Wei et al., 2009). P123/F127 mixture CMC value is shifted toward the CMC of pure P123, since P123 has higher CMC compared to F127 and present in the optimum mixture by high percentage (80%).

#### TEM

**Figure 2.**
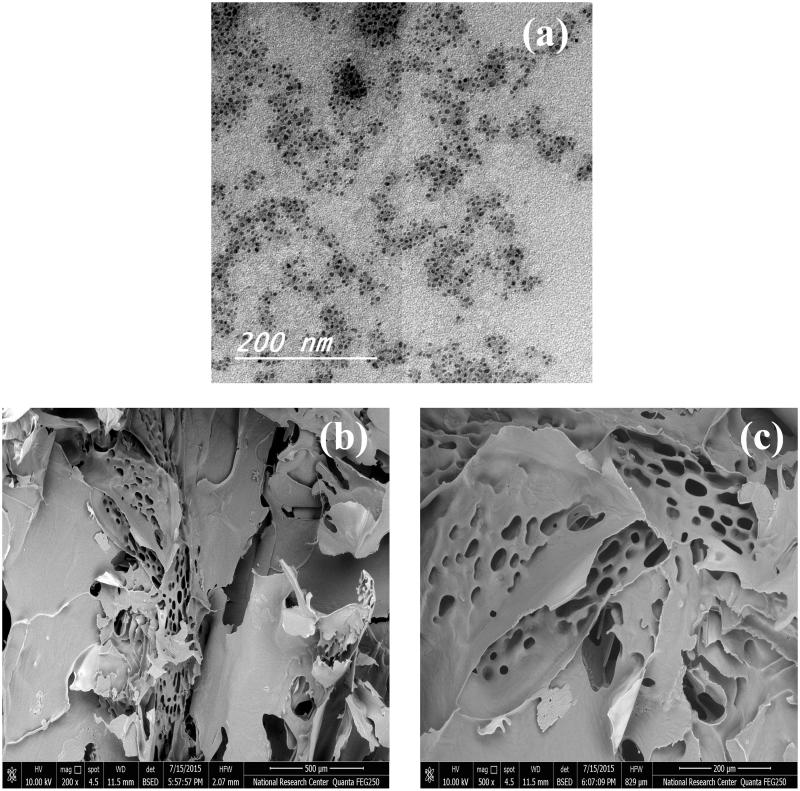
(a) TEM of optimum LCDP polymeric micelles formula, (b) and (c) SEM of optimum LCDP lyophilized polymeric micelles formula, magnification 200× and 500×, respectively.

Transmission electron microscopy images of LCDP polymeric micelles optimal formula represented in Figure 2(a), showed small-sized spherical polymeric micelles with uniform PS and homogenous distribution in aqueous medium. The observed PS in TEM images was in good agreement with that measured by the dynamic light scattering technique.

#### SEM

Scanning electron microscopy micrographs of LCDP polymeric micelles optimal formula represented in Figure 2(b,c), showed a highly porous structure for the lyophilized polymeric micelles. This highly porous structure could help in the rapid reconstitution of the lyophilized micelles to form the original LCDP polymeric micelles dispersion.

#### Saturation solubility in distilled water and 0.1 M HCl (pH = 1.2)

The saturation solubility of LCDP was found to be 0.0016 ± 0.19E-3 and 0.0021 ± 0.16E-3 mg/mL in water and 0.1 M HCl, respectively. While the saturation solubility of lyophilized LCDP suspension was 0.0024 ± 0.11E-3 and 0.0035 ± 0.27E-3 mg/mL in water and 0.1 M HCl, respectively. On the other hand, the saturation solubility of lyophilized LCDP polymeric micelles formula was found to be 0.726 ± 0.16 and 0.943 ± 0.19 mg/mL in water and 0.1 M HCl, respectively.

The saturation solubility of lyophilized LCDP polymeric micelles was approximately 450 times that of raw LCDP and 300 times that of lyophilized LCDP suspension in both water and 0.1 M HCl. This enhancement in LCDP solubility can be attributed to the interaction between the drug and Pluronic molecules and to the greater number of micelles formed at higher Pluronic to drug ratio (Parmar et al., 2013). LCDP solubilization increases by increasing the P123 percentage due to the higher LCDP affinity to the hydrophobic Pluronic (Wei et al., 2009).

#### *In vitro* dissolution rate study

**Figure 3.**
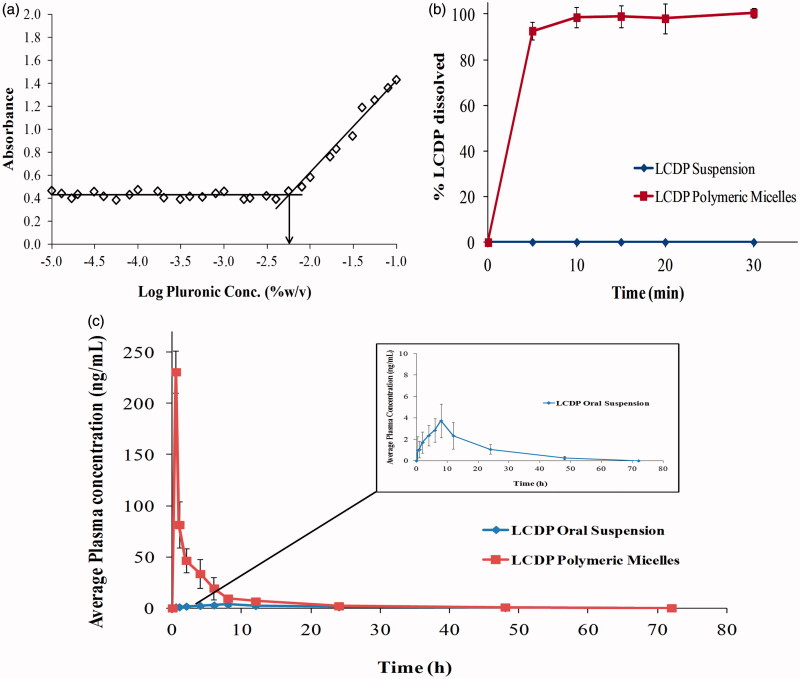
(a) Optimum Pluronic P123/F127 mixed polymeric micelles CMC determination by I_2_ UV spectroscopy method (b) *In vitro* dissolution rate profile of LCDP polymeric micelles in 0.1 M HCl (pH = 1.2) at 37 °C in comparison to raw LCDP and (c) The mean plasma concentration time curve after the administration of LCDP polymeric micelles and LCDP oral suspension to six albino rabbits.

*In vitro* LCDP dissolution rate profiles from the lyophilized LCDP polymeric micelles formula in comparison to that of LCDP suspension are illustrated in Figure 3(b). Lyophilized LCDP polymeric micelles showed significantly improved dissolution rate and extent in comparison to LCDP suspension. After 5 min, 92.69% of LCDP from the lyophilized polymeric micelles was dissolved in 0.1 M HCl. LCDP polymeric micelles showed complete dissolution after 30 min. On the other hand, LCDP suspension showed no dissolution in 0.1 M HCl along the 60 min.

The large increase in the saturated solubility of LCDP lyophilized polymeric micelles in 0.1 M HCl contributes greatly to the dissolution rate improvement. Upon addition to the dissolution medium, a polymeric micelles dispersion with enhanced LCDP solubility is formed, which enhances the dissolution of the poorly water soluble LCDP (Abdelbary and Makhlouf, 2014).

In addition, the hydrophilic F127 enhances the water penetration in the core of the micelles forming hydrophilic channels. The PEO corona of polymeric micelles may become more extended due to the interaction between the H^+^ and PEO chains of Pluronic copolymers, forming more hydrophilic channels in the PEO shell which increase the drug diffusion from the PPO core of the micelles. (Chen et al., 2013; Abdelbary and Makhlouf, 2014).

### *In vivo* bioavailability study in rabbits

Results obtained from the *in-vitro* dissolution rate study revealed that the dissolution rate of LCDP polymeric micelles formula was significantly improved compared to LCDP suspension. The optimum LCDP polymeric micelles formula was compared *in vivo* with oral LCDP suspension in six male healthy albino rabbits.

The LCDP average plasma-concentration time profiles following single oral dose administration of LCDP polymeric micelles and oral suspension are shown in Figure 3(c). LCDP polymeric micelles showed significantly higher *C*_max_ and AUC and lower *t*_max_ values relative to the LCDP oral suspension. The mean pharmacokinetic parameters obtained by non-compartmental analysis of the concentration–time data of LCDP after oral administration of the polymeric micelles and the oral suspension were given in Table 3. Statistical comparison between the pharmacokinetic parameters of LCDP polymeric micelles and oral suspension was shown in Table 3. Based on the average AUC_(0–∞)_, the relative bioavailability was found to be 685.69%.

**Table 3.** Mean pharmacokinetic parameters of LCDP following the administration of optimum LCDP polymeric micelles formula and LCDP oral suspension to six albino rabbits.

PK parameter	LCDP polymeric micelles	LCDP oral suspension	Statistical test
*C*_max_ (ng/ml)	230.68 ± 20.21	3.72 ± 1.55	*p* = .000
*t*_max_ (h)^a^	0.5	8	*p* = .000
AUC_(0–72)_ (ng h/ml)	493.22 ± 67.28	69.59 ± 28.38	*p* = .000
AUC_(0–∞)_ (ng h/ml)	498.87 ± 66.38	72.75 ± 26.97	*p* = .000
*λ*_z_ (h^−1^)	0.05 ± 0.01	0.05 ± 0.01	*p* = .791
*t*_1/2_ (h)	14.92 ± 3.21	15.02 ± 5.61	*p* = .976

^a^ Median.

In previous studies, Dinda and Pand (2014) reported a 1.5-fold increase in the bioavailability of LCDP nanosuspension compared to the commercial tablets, Geng et al. (2014) reported a 1.56-fold increase in the bioavailability of LCDP oral solid dispersion compared to the commercial oral tablets, Gannu et al. (2010) presented a 3.5-fold improvement in the bioavailability of LCDP after transdermal application of LCDP microemulsion gel in comparison to oral LCDP suspension and ElKasabgy et al. (2014) stated a five-fold increase in the bioavailability of LCDP from enteric coated capsules filled with lipotomes compared to the commercial oral tablets.

Based on the abovementioned results, it could be concluded that the significantly enhanced absorption and bioavailability of LCDP polymeric micelles, with a 6.85 fold increase in bioavailability than that obtained after administration of LCDP oral suspension, with higher *C*_max_ and shortened *t*_max_, could be due to the solubilizing effect of the polymeric micelles and the inhibition of the P-gp efflux by the Pluronic micelles which enhances the drug permeability and absorption to systemic circulation (Abdelbary and Makhlouf, 2014). In addition, the enhancement in extent of LCDP absorption may lead to reduction in the effect of CYP3A-mediated hepatic first pass metabolism by offering a higher fraction of the drug to pass to the systemic circulation leading to increased bioavailability.

These results suggest that promising polymeric micelles based LCDP dosage forms could be formulated with reduced therapeutic dose due to the enhanced LCDP bioavailability.

## Conclusions

Thin film hydration technique was successfully applied for the preparation of LCDP polymeric micelles with high EE% and small and uniform PS. Statistical analysis of the 3^2^ CCFD revealed that the quadratic model was the one of choice for EE%, PS and PDI. Model analysis showed that the Pluronic to drug ratio and P123 percentage had a significant effect on EE% and PS, while the PDI was significantly affected only by Pluronic to drug ratio. Optimal LCDP polymeric micelles formula with desirability 0.959 was chosen and evaluated. It had EE% of 99.23%, PS of 21.08 nm, and PDI of 0.11. The saturation solubility of the lyophilized LCDP polymeric micelles was approximately 450 times that of raw LCDP in both water and 0.1 M HCl. In addition, lyophilized LCDP polymeric micelles exhibited improved dissolution rate and extent in comparison to raw LCDP, showing complete dissolution after 30 min. The *in vivo* evaluation, in six male healthy albino rabbits, of optimum LCDP polymeric micelles formula revealed a 6.85-fold increase in bioavailability than that obtained after the administration of LCDP oral suspension. So it could be concluded that a promising polymeric micelles based LCDP dosage forms could be formulated and can provide an effective management of hypertension.
